# Effects of resveratrol on tolerance to ischemia/reperfusion injury in aged male mice: Role of autophagy and apoptosis

**DOI:** 10.1002/fsn3.3525

**Published:** 2023-06-28

**Authors:** Xiaogang Song, Chao Wei, Hui Huang, Xingdan Cao, Ziyi Chen, Yongqing Chen, Bing Wu

**Affiliations:** ^1^ Key Laboratory of Stem Cells and Gene Drugs of Gansu Province, Department of Cardiology The 940th Hospital of Joint Logistics Support Force of Chinese People's Liberation Army Lanzhou China; ^2^ Department of Neurology The First Medical Center of the Chinese People's Liberation Army General Hospital Beijing China; ^3^ Department of Geriatrics The 940th Hospital of Joint Logistics Support Force of Chinese People's Liberation Army Lanzhou China; ^4^ Department of Cardiology The Gansu Provincial Central Hospital Lanzhou China

**Keywords:** aged, apoptosis, autophagy, ischemia/reperfusion injury, resveratrol

## Abstract

Aged myocardium is more susceptible to ischemia/reperfusion (I/R) injury. Autophagy and apoptosis play important roles in cardiac I/R injury. However, whether resveratrol can reduce the I/R vulnerability of aged myocardium by regulating apoptosis and autophagy remains unclear. The present study aimed to investigate the effect of resveratrol on the tolerance to I/R injury in aged male mice and to determine the contribution of apoptosis and autophagy. We used aged C57 mice as our research subjects. The hearts of mice were isolated after 6 weeks of intragastric administration with resveratrol and subsequently perfused with Krebs–Henseleit buffer to produce the I/R model. We found that resveratrol alleviated cardiac I/R injury in aged mice, but not in SIRT1^+/−^ mice. Aged mice exhibited decreased LC3 and Beclin1 expressions, which were significantly rescued by resveratrol treatment. In addition, resveratrol decreased the expression of Bax and the activity of Caspase‐3, while increasing the expression of Bcl‐2 and the activity of SIRT1 in aged mouse hearts. Coimmunoprecipitation assays revealed that resveratrol facilitated the binding of Bax to Bcl‐2 and the dissociation of Bcl‐2 from Beclin1 in aged mouse myocardium. Conversely, SIRT1 knockout enhanced the formation of the Beclin1/Bcl‐2 complex and disrupted the interaction between Bcl‐2 and Bax. The above results indicate that resveratrol can reduce the vulnerability of myocardial I/R injury in senile myocardium by inhibiting apoptosis and upregulating autophagy through the SIRT1 signaling pathway.

## Graphical abstract

Autophagy and apoptosis play important roles in cardiac I/R injury. However, it remains largely unclear whether resveratrol can reduce the ischemic vulnerability of aged myocardium by regulating apoptosis and autophagy. The present study shows that resveratrol attenuates ischemic/reperfusion injury vulnerability of senile myocardium by inhibiting apoptosis and upregulating autophagy through SIRT1 signaling pathway.

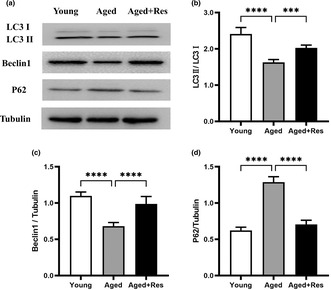

## 1 INTRODUCTION

The most effective treatment for acute ischemic heart disease is to restore blood flow to the ischemic myocardium as soon as possible. However, studies have found that with the reperfusion of blood flow, fatal damage occurs in the ischemic myocardium, which is called myocardial ischemia/reperfusion (I/R) injury (Cao et al., 2017). Patients often present with arrhythmias, no‐reflow phenomena, and cardiac dysfunction (Mokhtari‐Zaer et al., 2018). The pathogenesis of I/R injury may be related to oxidative stress, inflammatory response, calcium overload, mitochondrial dysfunction, apoptosis, and other factors. However, the specific mechanism remains to be further investigated.

Heart function deteriorates with age at a rate of 1% per year and the incidence of myocardial ischemia/reperfusion injury is rising as the population ages (Kane & Howlett, 2016). There are structural and functional differences between the aging heart and the younger heart, which may make the aging heart more susceptible to I/R injury (Boengler et al., 2009). Myocardial ischemia/reperfusion therapy has made great developments in recent years due to advances in drugs and surgery. However, elderly patients have poor tolerance of I/R injury, severe impairment of cardiac function, and a high incidence of adverse cardiovascular events and mortality. Therefore, the exploration of new drugs and therapeutic targets remains imperative for the treatment of myocardial I/R injury.

Our previous study has demonstrated that enhanced myocardium aldehydic toxicity and autophagy deficiency may contribute to heart senescence (Wu et al., 2016). Moreover, the increased apoptosis of cardiomyocytes in the aged heart aggravates the susceptibility to myocardial I/R injury (Wang et al., 2013). Based on these findings, this study speculated that the restoration of autophagy and the inhibition of apoptosis could alleviate myocardial I/R injury in naturally aged mice.

Resveratrol (Res) is a natural polyphenol present in a variety of plant species (Li, Tan, et al., 2022) and has potential beneficial effects on various cardiovascular diseases, such as atherosclerosis, hypertension, stroke, myocardial infarction, and heart failure (Li et al., 2012). Recent studies have shown that the most important properties of resveratrol are antioxidant and anti‐inflammatory (Pastor et al., 2019). By activating the AMPK/p38/Nrf2 signaling pathway, resveratrol can inhibit oxidative stress, reduce the inflammatory response, and protect cardiomyocytes (Xu et al., 2022). In addition, resveratrol partly reverses the decline of autophagy in the chronically ischemic myocardium (Sabe et al., 2014), which induces autophagy through activation of the AMPK/SIRT1 signaling pathway to maintain mitochondrial and cellular function (Breuss et al., 2019). Furthermore, it has been shown that long‐term application of resveratrol can significantly prevent I/R injury by inhibiting mitochondria‐mediated apoptosis (Liao et al., 2015). However, whether resveratrol plays a role in cardiac health during aging has not been clearly defined. In this respect, the purpose of this study was to investigate the role of resveratrol in I/R injury in the aged heart and to elucidate the mechanisms underlying autophagy and apoptosis.

## 2 MATERIALS AND METHODS

### 2.1 Animals

Male C57BL/6 mice (4 and 22 months) were purchased from the animal center of the 940th Hospital of Joint Logistics Support Force of Chinese People's Liberation Army. All experiments were approved by the Ethics Committee of Animal Experiments of the 940th Hospital of Joint Logistics Support Force of Chinese People's Liberation Army (No.2020KYLL132) and carried out in accordance with the Guidelines for the Care and Use of Experimental Animals issued by National Institutes of Health. SIRT1 KO mice (SIRT1^+/−^) were bred in our laboratory, and only male mice were used. Before the experiments, all the mice were fed with the same standard diet and sufficient water, with 12 h of alternating light and darkness for at least a week, so that they could acclimatize to their surroundings. There were three groups in the experiment, the young group, the aged group, and the aged+res group. Ten 4‐month‐old mice were taken as the young group. Twenty 22‐month‐old mice were divided into two groups, 10 in each group, as the aged group and the aged+res group using the random number table method.

### 2.2 Treatment with resveratrol

A quantity of 100‐mg resveratrol powder was fully dissolved with 100 mL of 25% alcohol to obtain 1 mg/mL resveratrol solution, which was divided and frozen in the refrigerator at −20°C and then dissolved at room temperature before use. The mice in the aged+res group were given resveratrol by gavage (2.0 mg/kg/d) once a day for 6 weeks according to their body weight (Sigma #501‐36‐0) (Liao et al., 2015). The mice in the aged group and the young group were given 20% alcohol by gavage (2.0 mg/kg/d) once a day for 6 weeks according to their body weight.

### 2.3 SIRT1 activity assay

SIRT1 deacetylase activity was measured in the crude nuclear extract from heart samples according to the manufacturer's instructions. Trichostatin A (0.2 mM; Sigma‐Aldrich), components of SIRT1 Fluorescent Activity Assay/Drug Discovery Kit (Enzo Life Sciences), including a fluorogenic acetylated Lys^382^ of p53 peptide (100 μmol/L) and NAD^+^ (170 μmol/L) at 37°C for 1 h, and then incubated in the developer at room temperature for 15 min. The fluorescent signal was measured with a fluorescence plate reader (excitation: 360 nm, emission: 460 nm).

The developer II solution was incubated with 2 mmol/L NAM and then mixed with the substrate to obtain the no‐enzyme and time‐zero negative control group. The activity of SIRT1 was calculated by correcting the fluorescence units of the measured samples and the no‐enzyme and time‐zero control group. The results were shown as fluorescent units relative to the control group. All of the reactions were performed in triplicate.

### 2.4 Western blotting and immunoprecipitation

Immunoblots were performed as previously described (Wu et al., 2016). The antibodies against Bax (#5023), Bcl‐2 (#3498), Beclin1 (#3495), LC3B (#3868), and P62 (#5114) were bought from Cell Signaling Technology. The antibodies against Tubulin (#ab179513) and β‐actin (#ab8226) were bought from the Abcam Company. Antibody fixation was tested by highly sensitive enhanced chemiluminescence (Millipore) and scanned with a Gel imaging system (Bio‐Rad Company). The gray values of the immunoblotting strips were analyzed by the image analysis software ImageJ (Fiji). Mix cell lysates with primary antibodies for immunoprecipitation analysis. Immunoprecipitation was performed by the following procedure. First, the target protein antibodies were mixed with the total cell protein to form the immunocomplexes. Second, the immunocomplexes were separated by SDS‐PAGE. Finally, western bolt was used for identification.

### 2.5 Caspase‐3 assay

The activity of Caspase‐3 was measured as previously described (Ma et al., 2009). First, 80 μl of precooled cell lysis buffer (Solarbio Life Science Company #R0030) was added to myocytes and laid on the ice for half an hour. Then, 35 μl of reaction buffer and 10 μl of Caspase‐3 colorimetric specific substrate (Ac‐DEVD‐pNA) were added together and incubated at 37°C for 1 h. Finally, the OD value at 405 nm was measured by a microplate reader and the Caspase‐3 activity was in positive proportion to the OD value.

### 2.6 Mouse heart perfusion

With a tail vein injection of heparin (110 U/kg) 10 min later, the mice were anesthetized by intraperitoneal injection of pentobarbital sodium (50 mg/kg). After successful anesthesia, the hearts were quickly removed through a midsternal incision and placed in Krebs–Henseleit buffer (118 mM NaCl, 4.7 mM KCl, 1.2 mM KH_2_PO4, 1.2 mM MgSO_4_, 25 mM NaHCO_3_, 2.5 mM CaCl_2_, 11 mM glucose) at 4°C. The hearts were mounted on a cardiac perfusion system (Shanghai Instrument Equipment Company) and perfused (4 mL/min) with a 95% oxygen and 5% carbon dioxide Krebs–Henseleit buffer (37°C, pH 7.4) at a constant pressure of 80 cm H_2_O. A custom‐made latex balloon was inserted into the left ventricle through the ascending aorta to stabilize the left ventricular end‐diastolic pressure at 5–10 mmHg. Firstly, the hearts were equilibrated in Krebs–Henseleit buffer for 30 min. Secondly, the hearts were perfused with a modified Krebs–Henseleit buffer (37°C, pH 6.8) containing 95% N_2_ and 5% CO_2_ without glucose. Finally, the hearts were then perfused with Krebs–Henseleit buffer (37°C, pH 7.4) containing 95% O_2_ and 5% CO_2_. The ischemia/reperfusion models were prepared using this process. The LabChart8 software (AD Instruments) was used to obtain the heart rates and the left ventricular pressures of the isolated hearts.

### 2.7 Statistical analysis

All data presentations are presented as mean ± standard deviation ($\bar{x}$ ± SD). Student's t‐test was used to analyze the significance of differences between the two groups. The significance of differences for more than two groups was analyzed with one‐way analysis of variance (ANOVA), followed by Tukey's post hoc test. Statistical analysis was performed by GraphPad Prism 9.0 (GraphPad Software, Inc.). *p* < .05 was considered as a statistically significant difference.

## 3 RESULTS

### 3.1 Resveratrol regulated the expression of Bax and Bcl‐2 in aged mouse hearts

**FIGURE 1:**
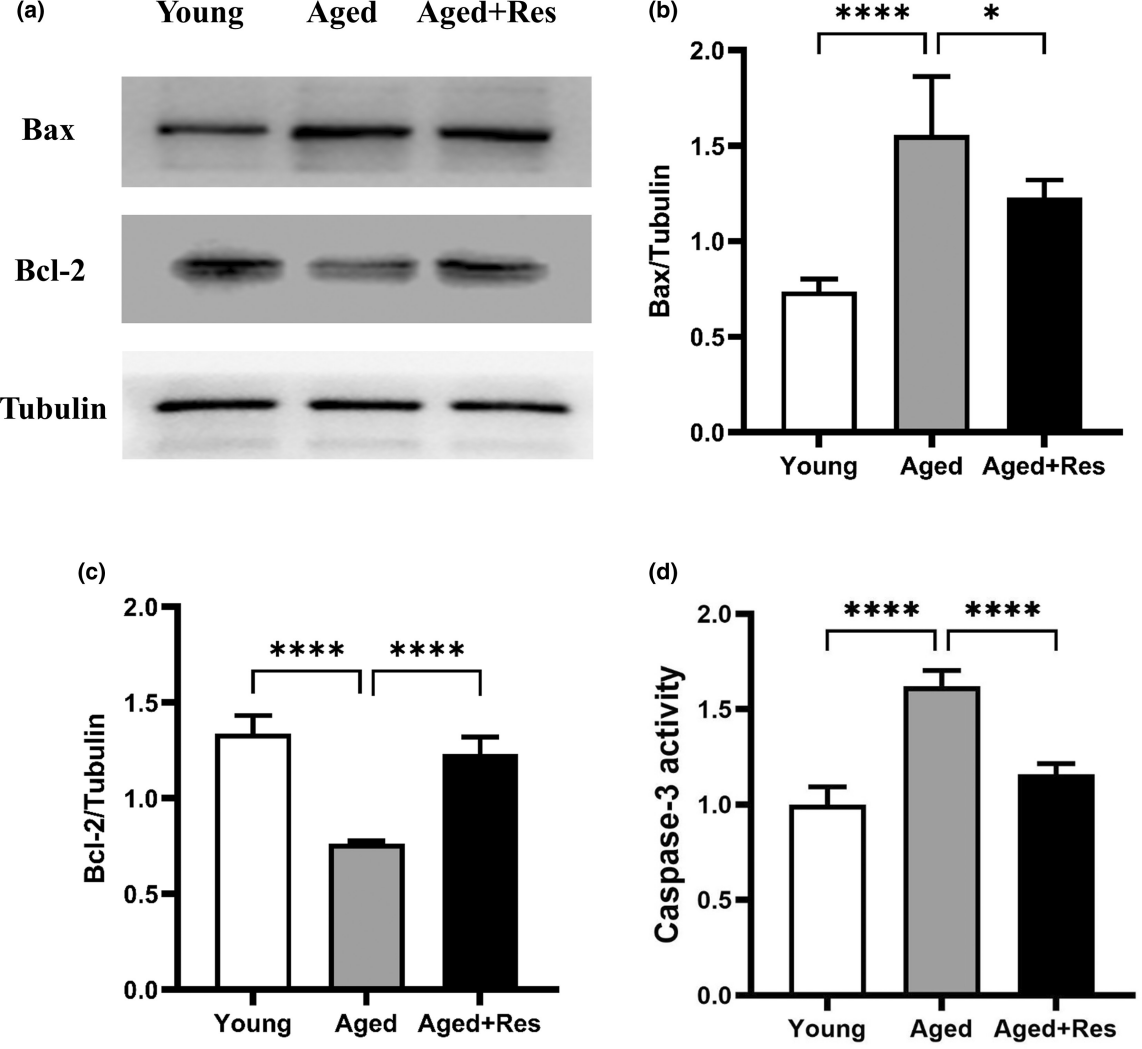
Resveratrol downregulated Bax expression and Caspase‐3 activity, and upregulated Bcl‐2 expression in aged mouse hearts. (a) Representative western blot images of Bax and Bcl‐2. (b) The quantitative analysis of Bax/β‐Actin. (c) The quantitative analysis of Bcl‐2/β‐Actin. (d) The quantitative analysis of Caspase‐3 activity relative to the young group. (mean ± SD *n* = 10 per group. *p < .05; **p < .01; ***p < .001; ****p < .0001).

Among critical regulators in cell apoptosis, Bax functions as a pro‐apoptotic while Bcl‐2 serves as an antiapoptotic factor (Guo et al., 2015). Therefore, we explored whether these two apoptotic factors were involved in resveratrol‐suppressed apoptosis. We found that resveratrol treatment significantly downregulated the expression of Bax, while upregulated the expression of Bcl‐2 in aged mouse hearts (Figure 1a–c). The Caspase‐3 activity assay was then used to further explore the role of resveratrol in the apoptosis of mice hearts. Our results showed that resveratrol treatment could partially inhibit the Caspase‐3 activity in aged mouse hearts (Figure 1d). Together, these results demonstrated that resveratrol may suppress apoptosis in aged mouse hearts through the Bcl2/Bax‐Caspase‐3 pathway.

### 3.2 Resveratrol promoted cardiac autophagy in aged mouse hearts

**FIGURE 2:**
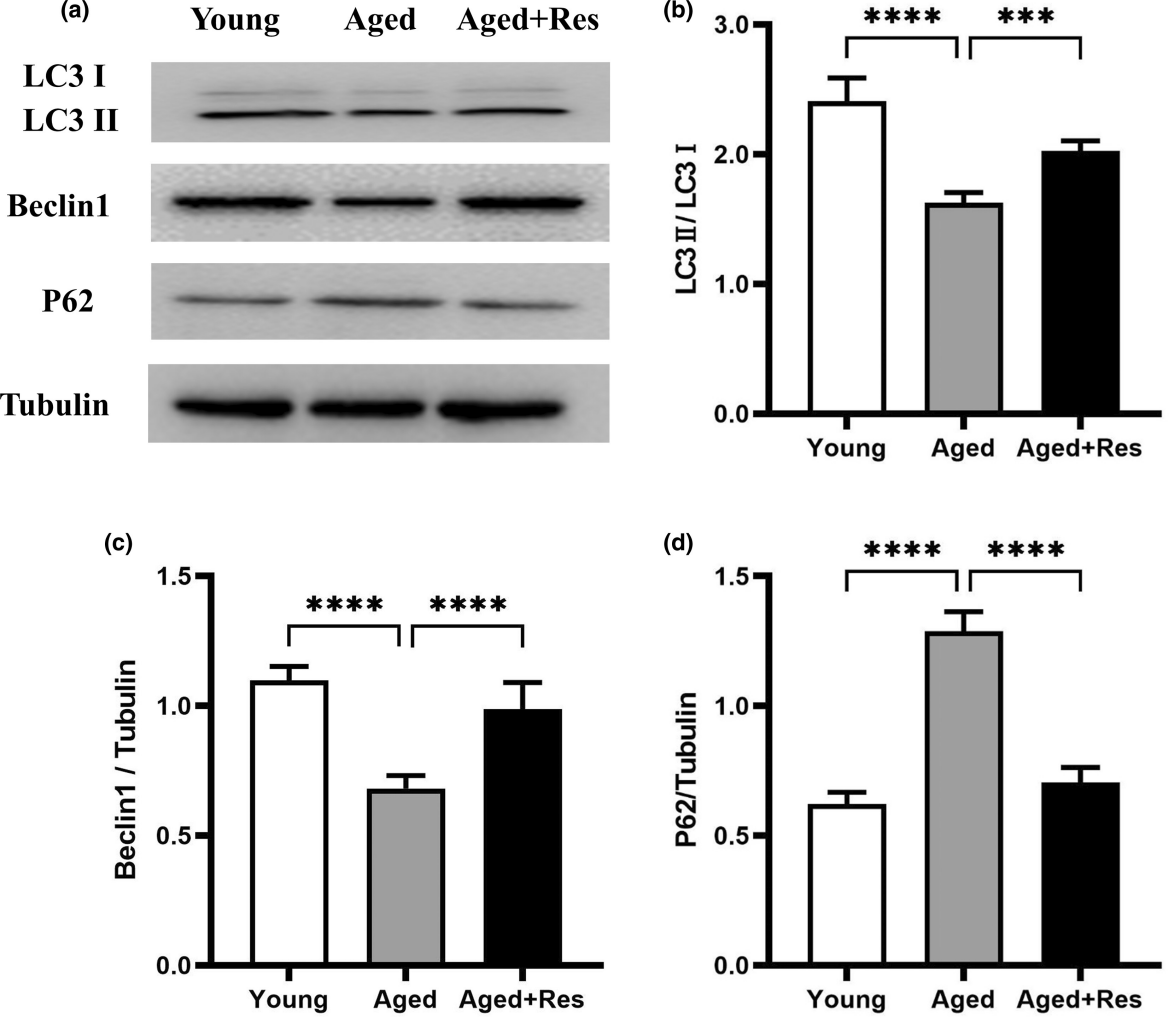
Resveratrol increased LC3 and Beclin1 expression, while downregulated P62 expression in aged mouse hearts. (a) Representative western blots images of LC3, Beclin1, and P62. (b) The quantitative analysis of LC3 II/LC3 I. (c) The quantitative analysis of Beclin1/Tubulin. (d) The quantitative analysis of P62/Tubulin. (mean ± SD *n* = 10 per group. *p < .05; **p < .01; ***p < .001; ****p < .0001).

Autophagic activity declines with age and may increase the risk of arrhythmias, reduce systolic function, and decrease myocardial tolerance to ischemia/reperfusion injury (Linton et al., 2015). To investigate the role of resveratrol in autophagy during the aging process, we monitored autophagy‐related protein expression in aged mouse hearts. Western blotting analysis showed elevated Beclin1 and LC3 II generation and decreased P62 expression in aged hearts treated with resveratrol (Figure 2a–d), suggesting that resveratrol improved autophagic activity in aged hearts.

### 3.3 Resveratrol treatment improved the tolerance of aged mouse hearts to I/R injury

**FIGURE 3:**
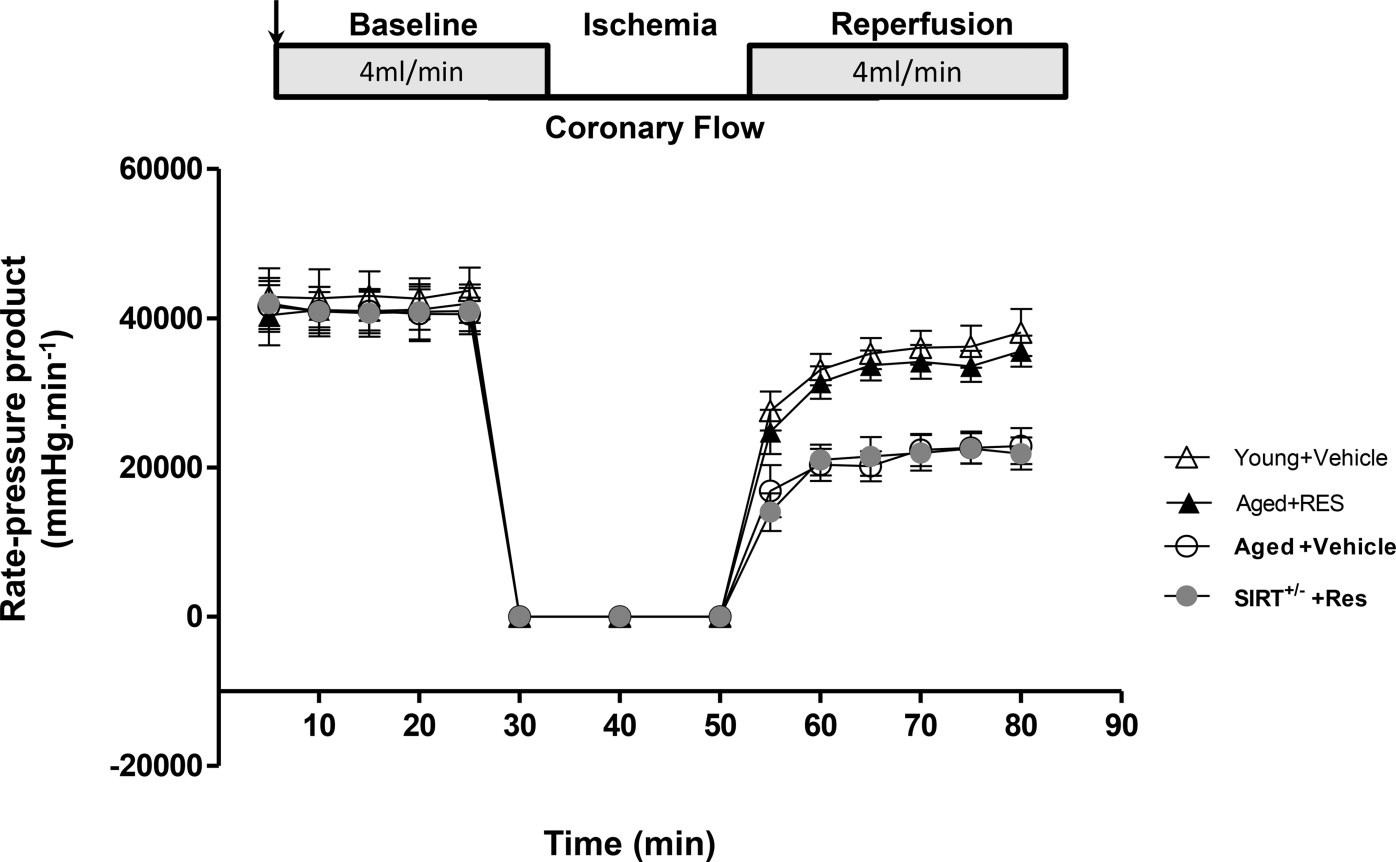
Resveratrol treatment protected heart function from deterioration in aged mice during I/R. Ex vivo heart perfusion (Langendorff) in Sirt1^+/−^ and Sirt1^+/+^ (wild‐type) mice. The determination of heart rate times left ventricular pressure during baseline perfusion, coronary flow cessation, and postischemia reperfusion treated with vehicle or resveratrol was obtained to reflect systolic function. The systolic function was impaired more seriously in aged hearts (*p* < .05 vs. young); resveratrol significantly restored the systolic function in aged hearts (*p* < .05 vs. Aged); the protective effect of resveratrol on Sirt1^+/−^ mice hearts disappeared (*p* < .05 vs. Sirt1^+/+^) (mean ± SD *n* = 10 per group).

To study the protective effect of resveratrol on I/R injury in aged mouse hearts, the isolated hearts were subjected to ischemia for 30 min and then reperfusion for 4 h to evaluate the changes in myocardial systolic function. Through in vitro heart perfusion, the systolic function was impaired after ischemia–reperfusion in both aged and young hearts, especially the aged hearts (Figure 3). Interestingly, resveratrol improved the recovery of systolic function after ischemia–reperfusion in aged hearts but not in Sirt1^+/−^ hearts. These findings suggest that the cardioprotective effect of resveratrol against I/R injury in aged mouse hearts may be performed through SIRT1, while SIRT1 deficiency disenabled the protective effect of resveratrol.

### 3.4 Resveratrol enhanced SIRT1 activity in aged mouse hearts

**FIGURE 4:**
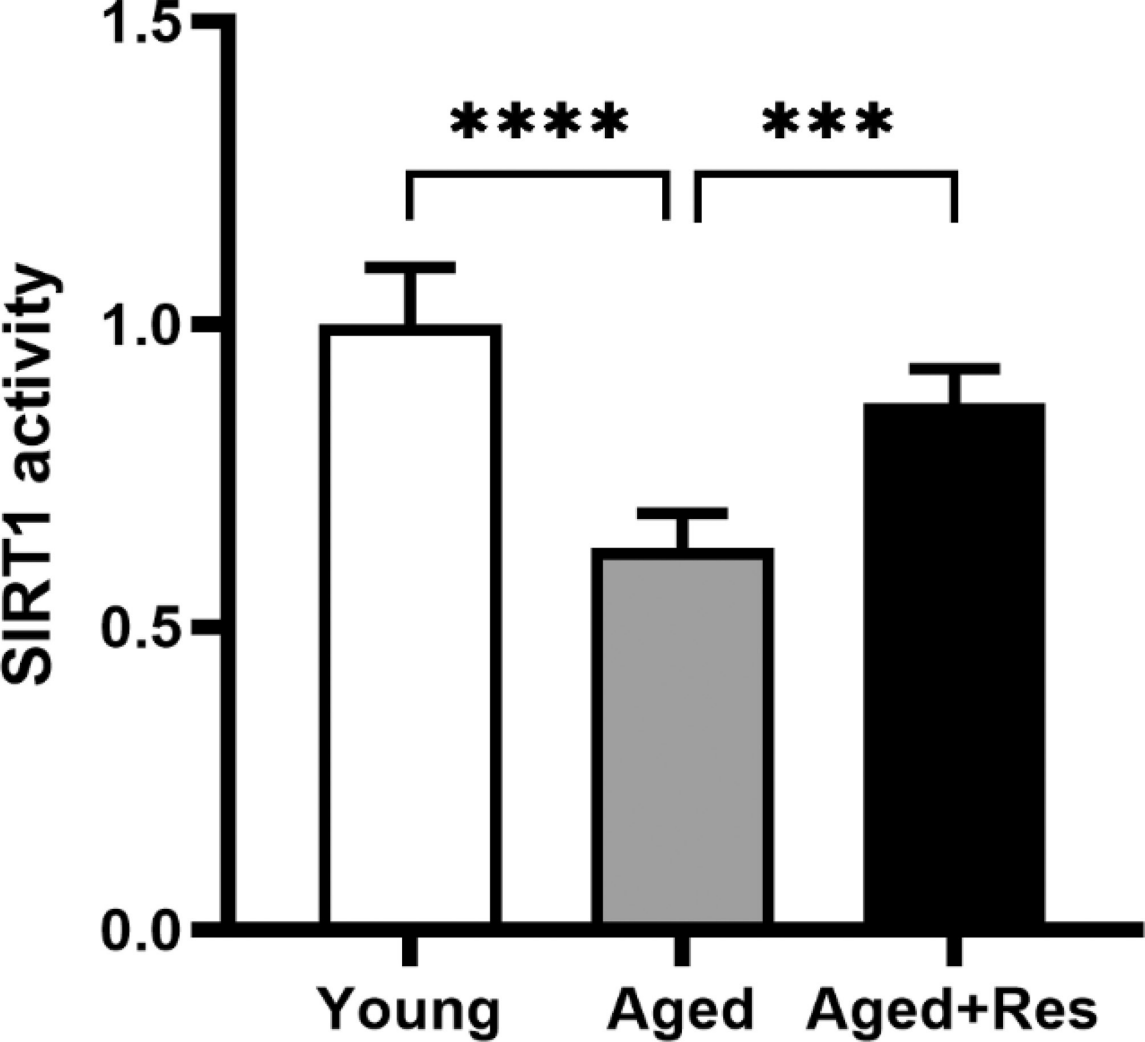
Impaired SIRT1 activity in aged mouse hearts. SIRT1 activity decreased in the aged group but increased significantly after resveratrol intervention. (mean ± SD *n* = 10 per group. *p < .05; **p < .01; ***p < .001; ****p < .0001).

SIRT1 plays an important role in the regulation of aging and age‐related cardiovascular diseases. However, a decline in Sirt1 activity in mice hearts with aging may contribute to the age‐related loss of cardioprotection. Resveratrol, an activator of SIRT1, upregulated SIRT1 activity in aged hearts (Figure 4).

### 3.5 Resveratrol did the deacetylation of Beclin1, thereby inhibiting the Beclin1–Bcl‐2 complex

**FIGURE 5:**
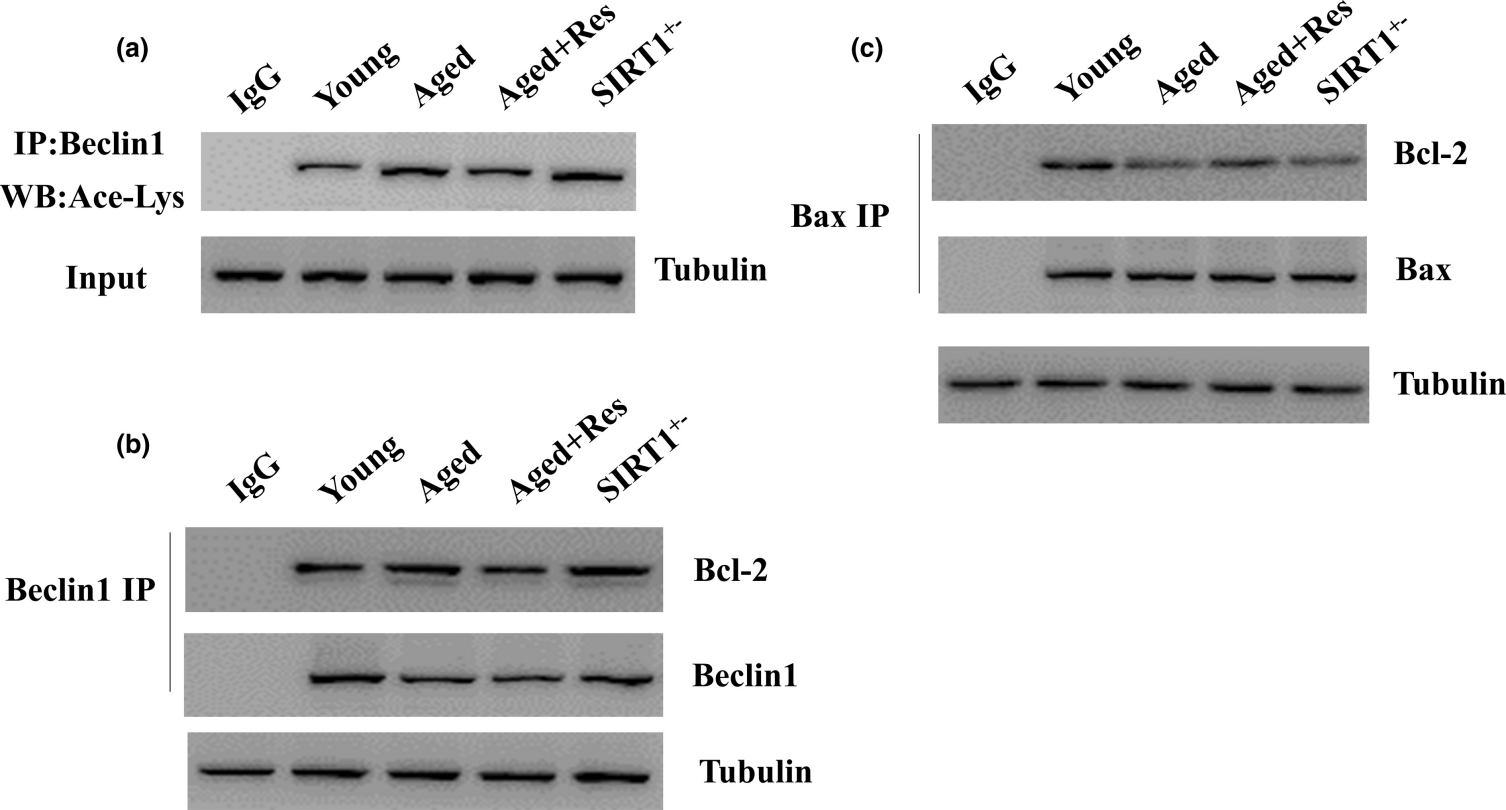
(a) Aging increased beclin1 acetylation but resveratrol significantly attenuated the beclin1 acetylation in aged mouse hearts but not in Sirt1^+/−^ hearts. (b) Aging enhanced the association between Beclin1 and Bcl‐2, but resveratrol dissociated Beclin and Bcl‐2 in aged mouse hearts. (c) Aging disrupted the association between Bax and Bcl‐2, whereas resveratrol treatment promoted the formation of Bax and Bcl‐2 complex in aged mouse hearts, but not in Sirt1^+/−^ hearts.

To elucidate how resveratrol promotes autophagy and inhibits apoptosis, we investigated how resveratrol interacted with beclin1 acetylation, Bcl‐2/Beclin1 complex, and Bcl‐2/Bax complex in aged mouse hearts by using immunoprecipitation. Resveratrol significantly attenuated the beclin1 acetylation (Figure 5a), inhibited the binding between Beclin1 and Bcl‐2(Figure 5b), and promoted the binding between Bcl‐2 and Bax (Figure 5c), but not in Sirt1^+/−^ hearts. These results suggest that resveratrol increases SIRT1 activity in aged mouse hearts and consequently induces interdependent modulation of Beclin1, Bcl‐2, and Bax, thereby simultaneously regulating autophagy and apoptosis.

## 4 DISCUSSION

The incidence of ischemic heart disease increases with age, even in people without major disease risk factors. Additionally, the mortality of ischemic heart disease also increases with age (Faber et al., 2011). Meanwhile, the incidence and mortality of I/R injury also significantly increase in the elderly population. However, the mechanisms of cardiovascular vulnerability in aging remain unclear.

Resveratrol is a kind of polyphenol drug extracted from natural plants. It is abundant in grapes, mulberries, and other plants, and has obvious antioxidant, antiaging, and antiatherosclerosis effects (Li et al., 2019). In a rat model of renovascular hypertension, resveratrol, independent of captopril, had a significant protective effect on cardiomyocyte remodeling and fibrosis in rats with renovascular hypertension (Restini et al., 2022). In another experiment, H9c2 cardiomyocytes were treated with rotenone to establish an I/R injury model. Resveratrol inhibited the expression of Bax by upregulating the vascular endothelial growth factor B (VEGF‐B), which, in turn, attenuated myocardial I/R injury (Yang et al., 2016). Numerous studies have shown that resveratrol has cardioprotective effects and antimyocardial I/R injury.

Silencing information regulator 2 related enzyme 1 (sirtuin1, SIRT1) is an important intracellular deacetylase, which attenuates doxorubicin‐induced cardiomyocyte apoptosis in mice through SIRT1‐mediated deacetylation of p53 (Zhang et al., 2011). In addition, SIRT1 is also a major autophagy regulator, which can reduce the acetylation of autophagy‐related proteins such as Atg5, Atg7, and Atg8 (Lee et al., 2008), and plays an important role in starvation‐induced autophagy by deacetylating FOXO1 and upregulating Rab7 (Hariharan et al., 2010; Li, Zheng, et al., 2022). Resveratrol exerts cardioprotective effects by increasing the expression of SIRT1 (Fourny et al., 2019). However, we observed decreased activity of SIRT1 in senescent cardiomyocytes, while the increased activity of SIRT1 upon resveratrol administration in aged mouse myocardium.

Autophagy is the progress of lysosome degradation of damaged, degenerated, or senescent proteins and organelles in cells, which can balance intracellular synthesis and catabolism, stabilize the intracellular environment, reduce stress injury of cardiomyocytes, and protect cardiac function. During I/R injury, appropriate activation of autophagy can increase cardiac ischemic tolerance, reduce cardiomyocyte injury, inhibit cardiomyocyte apoptosis, and protect cardiac function. Resveratrol can restore cardiac function in diabetic mice by regulating autophagy (Qu et al., 2019). In another hypoxia/reoxygenation model made by neonatal rat cardiomyocytes, resveratrol induced mitochondrial autophagy through activation of the SIRT1/SIRT3‐FoxO signaling pathway, which alleviated the hypoxia/reoxygenation injury of cardiomyocytes (Zheng et al., 2022). In the aged heart, elevated levels of oxidative stress, mitochondrial dysfunction, inadequate energy supply to cardiomyocytes, and increased misfolded and degenerated proteins are observed (Sciarretta et al., 2018). However, many features of age‐related cardiac dysfunction may be caused by the decline in autophagic activity with age. Increased autophagy function of the aging myocardium may be a potential therapeutic target (Linton et al., 2015). Beclin1 and LC3 II play an important role in autophagy, and their expression levels respond to the process of autophagy (Maejima et al., 2016; Wani et al., 2019). Previously, we found that the carbonylation of SIRT1 protein and autophagy in aged C57 mice were inactivated, while the acetylation of FoxO1 increased (Wu et al., 2016). In this study, we found that the expressions of Beclin1 and LC3 II and autophagy were decreased, while the ischemic vulnerability of aging myocardium was increased in the aging C57 mice. In addition, we also found that the decline in autophagy caused by the increased binding of Bcl‐2 to Beclin1 protein may contribute to the increased vulnerability of aging myocardial I/R. Moreover, resveratrol treatment reversed the above changes in a SIRT1‐dependent manner. After resveratrol treatment, the expression of SIRT1, Beclin1, and LC3 II in aged hearts increased. During ischemia–reperfusion, the elderly hearts showed obvious systolic dysfunction. In contrast, resveratrol showed significant improvement in cardiac systolic function. Resveratrol reduces I/R injury by increasing the autophagy of cardiomyocytes in aged C57 mice.

Autophagy and apoptosis have some common stimulators and regulatory proteins, which are controlled by the same upstream signals within the cells and can be interconverted under certain conditions. The dynamic balance between autophagy and apoptosis is crucial for cell function. When autophagy is insufficient, the autophagy‐apoptosis balance is broken and the cell becomes over‐apoptotic, which may accelerate the process of aging, especially aggravating I/R injury (Fu et al., 2021; Maiuri et al., 2007).

With the increase of age, mitochondrial damage and dysfunction lead to increased apoptosis. The Caspase and Bcl‐2 families play a critical role in the development of apoptosis. In this experiment, we found that the expression of Bax and the activity of Caspase‐3 increased significantly, while the expression of Bcl‐2 decreased markedly in aged mouse hearts. It may be one of the mechanisms underlying apoptosis in aged myocardial cells. Increased myocardial apoptosis reduces the ability of the aging heart to resist I/R injury. In an I/R model made by ligating the left anterior descending artery of the mouse hearts, resveratrol exerted antiapoptotic effects by inhibiting STIMI‐induced intracellular calcium accumulation. Resveratrol reduced cardiomyocyte apoptosis, alleviated I/R injury, and improved cardiac function in mice (Xu et al., 2019). However, we found that resveratrol can affect the expression of apoptotic proteins in aged hearts, and may inhibit myocardial apoptosis through the Bcl2/Bax‐Caspase‐3 signaling pathway, thereby reducing the I/R vulnerability of the aged myocardium.

Bcl‐2 is an antiapoptotic protein on the inner membrane of mitochondria. The Bcl‐2/Bax binding inhibits the function of Bax in promoting the release of mitochondrial cytochrome C, which inhibits apoptosis. P62 protein is a junction protein, which is degraded as an autophagic substrate binding during autophagic degradation. When autophagy is blocked, the expression of the P62 protein is significantly upregulated. Beclin1 is a regulatory protein of autophagosome formation. Bcl‐2 binds to Beclin1 through the BH3 domain to form a Bcl‐2/Beclin1 complex that prevents Beclin1‐dependent autophagy and increases apoptosis (Chen et al., 2018; Liang et al., 1998). The acetylation of Beclin1 leads to a decrease in its activity, inhibits autophagosome maturation, reduces autophagy, and increases apoptosis (Del Re et al., 2019; Sun et al., 2015).

In the process of apoptosis, the caspase family is activated to cleave and degrade Beclin1 to diminish autophagy (Booth et al., 2020). Meanwhile, Caspase‐3 induces apoptosis by increasing endonuclease activity and causing nucleosome DNA lysis. Caspase‐3 is a key execution molecule of apoptosis. Therefore, activated Caspase‐3 is an important marker of apoptosis.

In aged hearts, Beclin1, Bcl‐2 expression, and SIRT1 activity were significantly downregulated, while Bax expression and Caspase‐3 activity were significantly upregulated. The autophagy–apoptosis balance was disrupted, resulting in increased I/R injury in aged hearts.

In this study, we found that resveratrol significantly attenuated the acetylation of Beclin1, inhibited the binding between Beclin1 and Bcl‐2, and promoted the binding between Bcl‐2 and Bax in wild‐type but not in SIRT1^+/−^ mice hearts. Secondly, we also found that resveratrol significantly upregulated LC3‐II/LC3‐Iratio and Beclin1 expression and decreased p62 expression by increasing SIRT1 activity in aged hearts of C57 mice. Finally, resveratrol increased the expression of Bcl‐2, decreased the expression of Bax and the activity of Caspase‐3, and significantly improved the aged heart function of C57 mice during ischemia/reperfusion. These results indicate that resveratrol attenuates apoptosis by inducing autophagy through the upregulation of SIRT1‐mediated deacetylation of Beclin1, ultimately reducing I/R injury and improving the tolerance of I/R injury in aged mouse hearts.

## 5 CONCLUSION

In summary, this study indicates that increased apoptosis and decreased autophagy in aging myocardium may contribute to increased vulnerability to I/R injury. Resveratrol could attenuate aging myocardium apoptosis and improve the tolerance of aged hearts to I/R injury by enhancing autophagy mainly in a SIRT1‐dependent manner.

## AUTHOR CONTRIBUTIONS

**Xiaogang Song:** Conceptualization (equal); Investigation (equal); Methodology (equal); Writing original draft (equal). **Chao Wei:** Investigation (equal); Data curation (equal); Methodology (equal); **Hui Huang:** Resources (equal); Data curation (equal); Validation (equal). **Xingdan Cao:** Visualization (equal); Validation (equal). **Ziyi Chen:** Visualization (equal); Validation (equal). **Yongqing Chen:** Conceptualization (equal); Writing review and editing (equal); Supervision (equal). **Bing Wu:** Conceptualization (equal); Writing review and editing (equal); Methodology (equal); Funding acquisition (equal); Project administration (equal).

## FUNDING INFORMATION

This study was supported by Scientific Research Project on Health Industry of Gansu Province, Grant/Award Number: GSWSKY2020‐13; Research Program of the 940th Hospital of Joint Logistics Support Force of Chinese People's Liberation Army, Grant/Award Number: 2021yxky038; Natural Science Foundation of Gansu Province, Grant/Award Number: 18JR3RA402; and Project of Northwest Minzu University, Grant/Award Number: 31920200012.

## CONFLICT OF INTEREST STATEMENT

The authors declare that they do not have any conflict of interest.

## ETHICS STATEMENT

This study was approved by the Ethics Committee of Animal Experiments of the 940th Hospital of Joint Logistics Support Force of Chinese People's Liberation Army (No.2020KYLL132).

## INFORMED CONSENT

Written informed consent was obtained from all study participants.

## Data Availability

The data can be submitted from the corresponding authors upon request.
